# Small interfering RNA targeting CDC25B inhibits liver tumor growth in vitro and in vivo

**DOI:** 10.1186/1476-4598-7-19

**Published:** 2008-02-12

**Authors:** Xinrui Yan, Mei-Sze Chua, Jing He, Samuel K So

**Affiliations:** 1 Asian Liver Center, Department of Surgery, Stanford University School of Medicine, Stanford, CA 94305, USA; 2 Department of Surgery and Asian Liver Center, 300 Pasteur Drive, H3680, Stanford CA 94305-5655, USA

## Abstract

**Background:**

Using gene expression profiling, we previously identified CDC25B to be significantly highly expressed in hepatocellular carcinoma (HCC) compared to non-tumor liver. CDC25B is a cell cycle-activating phosphatase that positively regulates the activity of cyclin-dependent kinases, and is over-expressed in a variety of human malignancies. In this study, we validated the over-expression of CDC25B in HCC, and further investigated its potential as a therapeutic target for the management of HCC.

**Results:**

Quantitative real-time polymerase chain reaction and immunohistochemical staining of patient samples confirmed the significant over-expression of CDC25B in HCC compared to non-tumor liver samples (*P *< 0.001). Thus, intefering with the expression and activity of CDC25B may be a potential way to intervene with HCC progression. We used RNA interference to study the biological effects of silencing CDC25B expression in HCC cell lines (Hep3B and Hep40), in order to validate its potential as a therapeutic target. Using small oligo siRNAs targeting the coding region of CDC25B, we effectively suppressed CDC25B expression by up to 90%. This was associatetd with significant reductions in cell growth rate, cell migration and invasion through the matrigel membrane, and caused significant cell cycle delay at the G2 phase. Finally, suppression of CDC25B significantly slowed the growth of Hep40 xenografts in nude mice.

**Conclusion:**

Our data provide evidence that the inhibition of CDC25B expression and activity lead to suppression of tumor cell growth and motility, and may therefore be a feasible approach in the clinical management of HCC.

## Background

Hepatocellular carcinoma (HCC) is the primary adult malignancy of the liver, and is the fifth most frequent cancer in the world [1]. It causes significant mortality, especially in countries where there is a high prevalence of chronic hepatitis B virus and hepatitis C virus infection [2]. One of the reasons for this high mortality is that the tumor usually presents at a stage when curative surgery is no longer feasible because of intrahepatic or extrahepatic metastases. Therefore, more effective therapies need to be developed to treat HCC patients who are not surgical candidates.

All cancers share the common feature of a disordered cell cycle that is characterized by rapid and uncontrolled cell growth. Thus, targeting the molecules that regulate the cell cycle has become a major thrust in the development of anticancer therapeutics [3]. CDC25 phosphatases, as activators of the Cdk/cyclins, play critical roles in the regulation of the eukaryotic cell cycle. Because of their over-expression and correlation with poor prognosis in many diverse cancers, CDC25 phosphatases are attractive targets for anticancer drug development. The three human CDC25 isoforms (CDC25A, CDC25B, and CDC25C) are responsible for dephosphorylating Cdk/cyclins on pThr14 and/or pTyr15 residues. This dephosphorylation triggers the final activation of Cdk/cyclin activity during normal cell cycle progression [4,5]. CDC25A controls both the G1-to-S and G2-to-M transitions, whereas CDC25B and CDC25C are regulators of the G2-to-M transition. The activity of CDC25B is highly regulated by phosphorylation *via *several protein kinases (e. g. CK1, PKB/Akt, CDK/cyclin, p38, MAPKAP kinase 2, pEg3 and aurora A), which in turn regulate CDC25B activity, stability and/or subcellular localization [6-14]. Thus, at the same stage of the cell cycle, a large diversity of phosphorylated CDC25B molecules with different properties and functions may be generated *via *the activity of several independent kinases.

CDC25A and CDC25B, being oncogenes, are over-expressed in many different primary human cancers, such as head and neck cancer, poorly differentiated non-small cell lung cancer, advanced stage gastric cancer, non-Hodgkin's lymphomas, colon cancer, esophagus cancer, breast cancer, and ovarian cancer. [15-22]. Over-expressions of CDC25A and CDC25B are often associated with malignant features such as higher grade and more agrressive tumors, and poorer prognosis [23]. Xu *et al *recently reported the over-expression of CDC25A mRNA and protein in HCV-associated HCC tumors compared to paired non-tumor liver tissues. The over-expression of CDC25A was also associated with aggressive cancer phenotypes including portal vein invasion and dedifferentiated histology. In contrast, CDC25B was not over-expressed in HCV-associated HCC tumors, and was not associated with any clinicopathological parameters.

Our group previously studied the gene expression profiles in over 200 liver tissue samples [24], and identified CDC25B as one of most significantly over-expressed genes in HCC compared to non-tumor liver. We first validated the over-expression of CDC25B in HCC, at both the RNA and protein levels. Next, we used specific small inhibitory RNA oligos against CDC25B to study the biological effects of CDC25B suppression in HCC cell lines, especially in regard to cell proliferation, invasion, migration, and cell cycle. Our results suggest that CDC25B may be a potential diagnostic marker, as well as a therapeutic target for HCC.

## Results

### CDC25B is significantly overexpressed in hepatocellular carcinoma compared to non-tumor liver

**Figure 1:**
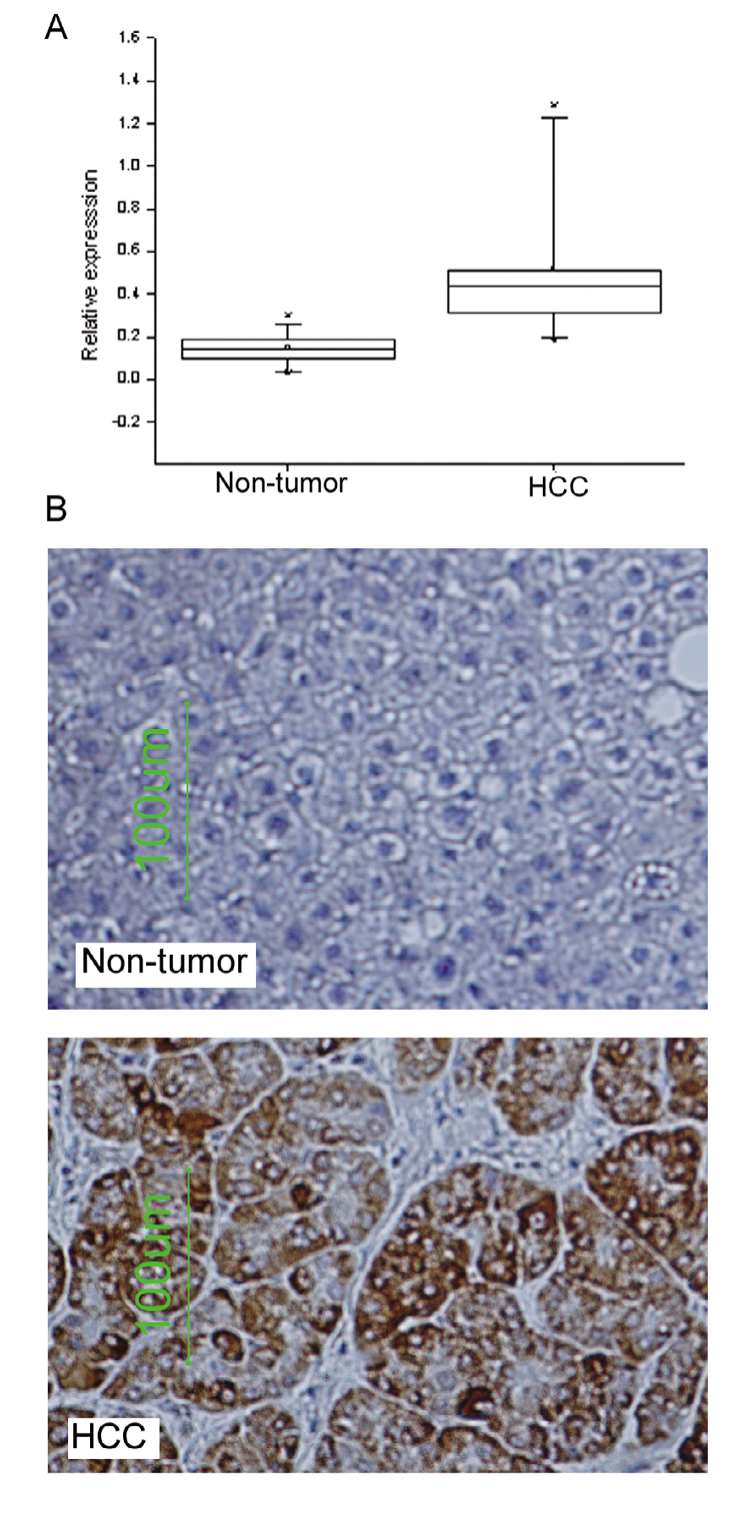
**CDC25B is over-expressed in hepatocellular carcinoma compared to non-tumor liver**. A) Significant over-expression of CDC25B transcript in hepatocellular carcinoma tumor compared to adjacent non-tumor liver in 24 pairs of tissue specimens (*P *< 0.001). The amount of CDC25B was normalized with 18s rRNA to control for RNA amount variation. B) Representative immunostaining of CDC25B in hepatocellular carcinoma tissue and adjacent non-tumor liver tissue. Photos were taken at ×20 magnification.

In an earlier gene expression study of hepatocellular carcinoma, CDC25B was upregulated in tumor compared to non-tumor samples. We used quantitative real-time PCR assays to validate the observed upregulation of CDC25B in hepatocellular carcinoma using 24 pairs of HCC tissues and adjacent non-tumor tissues. CDC25B was found to be expressed at significantly higher levels in HCC (median of 0.443) compared to adjacent non-tumors (median of 0.149) (*P *< 0.001) (Fig. 1A). Further statistical analyses showed no significant correlations between CDC25B transcript over-expression and clinicopathological features of the specimens.

Immunohistochemistry of liver tissue arrays further confirmed the over-expression of CDC25B protein in HCC tissues compared to non-tumor liver tissues (*P *< 0.001) (Table 1, Fig. 1B). Whereas only 1/42 (2.4%) non-tumor tissues showed greater than intermediate signal, 142/248 (57.3%) of HCC tissues showed intermediate or strong signal. Staining of CDC25B was observed in the cytoplasm. The lack of detailed clinical information from these archived pathology specimens precluded further analyses of associations between CDC25B protein over-expression and other clinicopathological features.

**Table 1:** CDC25B protein expression in human HCC and non-tumor liver tissues^a^

CDC25B protein expression score	HCC	Non-tumor
	(n = 248)	(n = 42)
0 (negative)	26 (10.5%)	24 (57.1%)
1 (weak signal)	80 (32.3%)	17 (40.4%)
2 (intermediate signal)	94 (37.9%)	1 (2.4%)
3 (strong signal)	48 (19.4%)	0

^a^Significantly higher in HCC compared to non-tumor liver when comparing scores 2 and 3 combined vs scores 0 and 1 combined (*P* < 0.001, Fisher's Exact Test).

### siRNA effectively suppresses CDC25B expression in Hep3B and Hep40 cells

To validate the potential of CDC25B as a therapeutic target for HCC, our approach was to use siRNA oligos to deplete CDC25B expression in HCC cells, and to study the biological effects of this suppression. Three CDC25B-specific siRNA (siRNA1-CDC25B, siRNA2-CDC25B, and siRNA3-CDC25B) and negative control siRNA-N (all at 50 nM) were transfected into Hep3B and Hep40 cells (2 tumorigenic cell lines that over-express CDC25B based on Western blot results). Western blotting confirmed suppression of CDC25B expression in CDC25B siRNA transfected cells, with greatest suppression by siRNA2-CDC25B. Cells transfected with siRNA-N or RNAiMAX alone had no effects on CDC25B expression (data not shown). We therefore chose to use siRNA2-CDC25B for all subsequent experiments.

**Figure 2:**
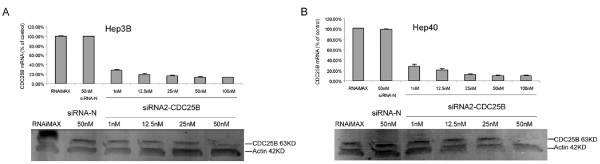
**SiRNA2-CDC25B reduces CDC25B expression in Hep3B and Hep40 cells**. A) Both CDC25B mRNA and expression levels were reduced dose-dependently by siRNA2-CDC25B in Hep3B cells. B) Both CDC25B mRNA and expression levels were reduced dose-dependently by siRNA2-CDC25B in HepG2 cells. Cells were transfected with siRNA2-CDC25B at different concentrations (1, 12.5, 25, 50 and 100 nM) for 48 hours and RNA extracted for analysis by quantitative real-time PCR as described under 'Materials and Methods'. Cell were harvested and lysed and Western blotting was performed as described. The level of CDC25B mRNA expression was normalized with that of human 18s RNA expression. The level of CDC25B mRNA in the untransfected cells were designated as 100%. Columns, mean of three independent experiments; bars, SD. Cells were harvested 48 hours after transfection and cell lysates detected for CDC25B and β-Actin using Western blot.

Transfection with siRNA2-CDC25B caused a dose-dependent decrease in CDC25B mRNA levels 48 hours post-transfection (Fig. 2), with greatest suppressions at 50 nM and 100 nM concentrations in both Hep3B (Fig. 2A) and Hep40 cells (Fig. 2B). At 50 nM siRNA2-CDC25B, mRNA levels were suppressed by up to 86% in Hep3B cell lines and 90% in Hep40 cell line by siRNA2-CDC25B. Since 100 nM did not cause greater suppression than 50 nM, we used 50 nM siRNA2-CDC25B for all subsequent experiments.

### Silencing of CDC25B inhibits proliferation, invasion and migration in Hep3B and Hep40 cells

**Figure 3:**
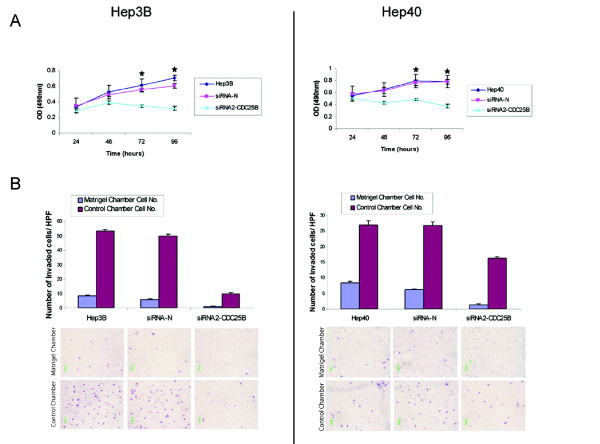
**Suppression of CDC25B expression by siRNA2-CDC25B inhibits cell proliferation, migration, and invasion**. A) The growth rates of siRNA2-CDC25B transfected and control cells were determined using the proliferation assay described under 'Materials and Methods'. The data were obtained from three independent experiments, with triplicates in each experiment. B) Invasion and migration of siRNA2-CDC25B transfected and control cells were assessed 48 hours post-transfection, using BD Biocoat Matrigel and control chambers respectively. Invaded or migrated cells were stained and counted (per high power field, HPF). Columns, mean of three independent experiments; bars, SD; *P < 0.01 for siRNA2-CDC25B *versus *siRNA-N transfected cells.

We next studied the effects of CDC25B suppression on the growth and invasive properties of HCC cell lines. Hep3B and Hep40 cells were transfected with siRNA-N or siRNA2-CDC25B, and cell growth was assessed daily over 4 days. Both cell lines transfected with siRNA2-CDC25B showed significantly slower growth rates than untransfected or siRNA-N transfected cells (*P *< 0.01 at 72 hours and 96 hours; Fig. 3A). This suggests that CDC25B mediates cell proliferation in HCC cells, and that its suppression led to growth inhibition (Fig. 3A).

Additionally, Hep3B and Hep40 cells transfected with siRNA2-CDC25B showed significantly reduced ability to migrate across non-Matrigel coated control membranes (*P *< 0.01; Fig. 3B) and to invade through Matrigel coated membranes (*P *< 0.01; Fig. 3B). After siRNA2-CDC25B transfection, migration of Hep3B and Hep40 cells were decreased by 4 and 2-folds respectively, and invasion were decreased by 4 and 5-folds respectively, relative to cells transfected with control siRNA-N. These findings indicate that silencing of CDC25B has significant inhibitory effects on cell invasion and migration.

### Silencing of CDC25B delays G2/M transition in Hep3B and Hep40 cells

**Figure 4:**
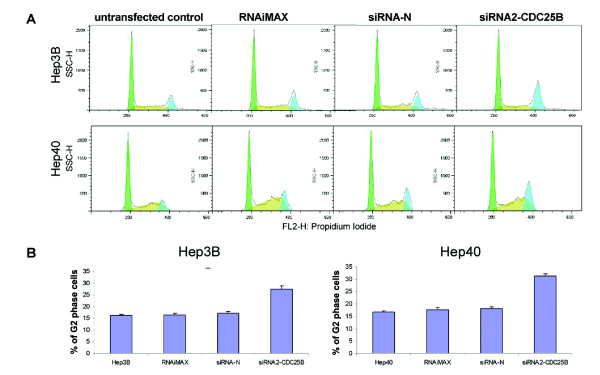
**Cells with reduced CDC25B level are delayed in G2/M transition**. Hep3B and Hep40 cells were transfected with siRNA2-CDC25B, siRNA-N or RNAiMAX for 48 hours. Cell cycle distribution was determined by flow cytometry. A. Representative set of histograms for transfected and control cells. B. Percentage of cells in G_2 _stage. Columns, mean of at least three independent experiments; bars, SD; *P *< 0.05 for siRNA2-CDC25B *versus *siRNA-N transfected cells.

To monitor the effect of CDC25B siRNA targeting on the cell cycle, we analyzed the DNA content of siRNA-transfected Hep3B and Hep40 cells by FACS at 48 hours after transfection. After siRNA2-CDC25B transfection, the percentage of cells in G2 or M phase was increased by 1.6 and 1.9 folds in Hep3B or Hep40 cells respectively (Fig. 4). Cells transfected with siRNA-N showed no changes in cell cycle distribution. The results demonstrated that RNAi-mediated reduction of CDC25B level leads to a delay in the G2/M transition.

### Silencing of CDC25B delayed tumor progression in a xenograft mode

**Figure 5:**
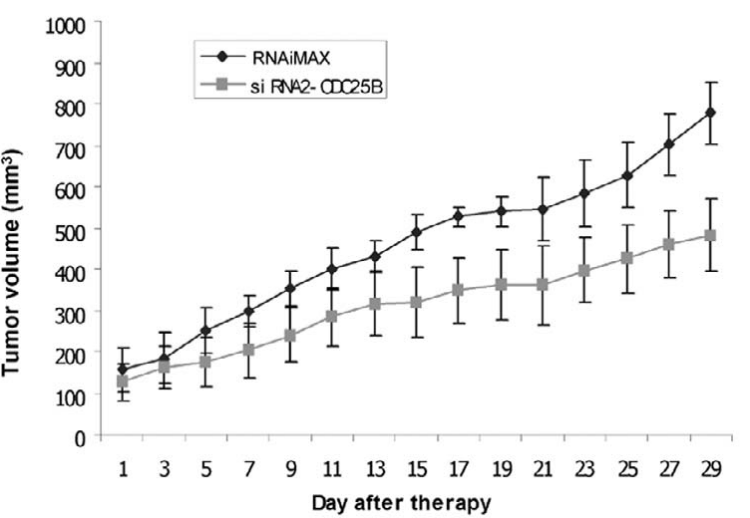
**Suppression of CDC25B expression by siRNA2-CDC25B delayed tumor progression in a xenograft model**. Hep40 cells were subcutaneously inoculated into the flanks of 10 mice (10^7 ^for each). When the tumor size reached an average of 200 mm^3^, 5 mice were given 8 μl RNAiMAX diluted to 30 μl with PBS, and another 5 mice given 5 μg siRNA and 8 μl RNAiMAX diluted to 30 μl with PBS by intratumor injection every two days. The tumor volume was assessed before each injection using a digital caliper. Significant differences in the tumor volumes of both groups were observed beginning from day 7 of siRNA2-CDC25B injection (*P *< 0.05). Columns, mean of the tumor size of five mice; bars, SD.

We next studied the *in vivo *effects of CDC25B suppression on tumor growth. Hep40 xenografts were grown in nude mice, and mice were given intratumoral injections of siRNA2-CDC25B and RNAiMAX (n = 5) or RNAiMAX alone (n = 5) every 2 days. All mice in the siRNA2-CDC25B group had a significantly smaller tumor size compared with that observed in RNAiMAX group (*P *< 0.05 from day 7 onwards, *P *< 0.01 from day 15 onwards) (Fig. 5).

## Discussion

We previously identified CDC25B mRNA to be significantly up-regulated in human HCC tumor compared to non-tumor liver tissues. Herein, we validated the significant over-expression of CDC25B transcript in HCC using quantitative real-time PCR, and of CDC25B protein using immunohistochemical staining of tissue arrays. Additionally, we found that the specific silencing of CDC25B expression (by up to 90%) in HCC cell lines inhibited *in vitro *cell proliferation, migration, and invasion, and also delayed HCC xenograft growth *in vivo*. Our data suggest that CDC25B, a key factor in regulating the cell cycle, may be a potential therapeutic target in HCC.

CDC25B is over-expressed in various types of human carcinomas, but the mechanism(s) regulating its expression remains unclear. Previous studies in colorectal [25], gastric [17], non-Hodgkin's [18] and ovarian cancers [22] showed that CDC25 over-expression was not due to gene amplification. It was originally shown that the transcriptional and catalytic activities of CDC25B are directly regulated by two other protooncogene products, c-raf and c-Myc, respectively [26,27]. As CDC25B contains functional binding sites for Myc/Max [27], many studies have looked for a correlation between c-Myc and CDC25 expression. Positive correlation has been detected in non-Hodgkin's lymphoma [18], neuroblastoma [28], non-small cell lung cancer [29]. In HBV associated HCC, HBx directly up-regulates Myc expression and induces hepatocarcinogenesis [30]. Additionally, chromosomal alterations frequently occur on chromosomes 1p, 6q, 8q and 13q in HCC, and encompass the amplification of c-Myc at 8q [31]. Thus, the over-expression of CDC25B in HCC could be related to the up-regulation of c-Myc. Alternatively, it has been suggested that post-translational modification leads to an enhanced stability of CDC25B [32]. CDC25B is an unstable protein whose proteasomal degradation is proposed to be controlled by beta-TrCP, but a splice variant of CDC25B (CDC25B2) is not degraded during mitosis, as are the other isoforms. In particular, up-regulation of the CDC25B2 variant was found in aggressive non-Hodgkin's lymphomas and in colorectal carcinoma [33,25]. This isoform may contribute to CDC25B up-regulation in human tumors.

As CDC25B promotes cell cycle progression and is over-expressed in numerous rapidly dividing cancer cells, CDC25B over-expression is expected to correlate with the rate of proliferation, consistent with our results that CDC25B siRNA suppressed proliferation of HCC cells. This inhibition of proliferation may result from the observed G2/M phase arrest caused by CDC25B siRNA. CDC25B over-expression was found to be significantly associated with a high proliferative activity of human non-hodgkin's lymphomas [18], and to enhance the proliferation of mammary epithelial cells in transgenic mice over-expressing human CDC25B [34]. CDC25B over-expression in tumors may help to circumvent many of the checkpoints that would otherwise hinder cell proliferation, and this lack of proper checkpoint control further contributes to the malignant nature of many tumors [35,36]. The correlation between CDC25B and c-Myc expression levels may also suggest that c-Myc contributes towards the proliferative activity of tumors with high CDC25B levels [18,37].

Our functional assays further suggest that CDC25B promotes cellular invasion and migration of HCC cells. Consistently, the over-expression of CDC25B in gastric cancer was reported to be associated with advanced stage and deep invasion [17], and also with high rates of lymphatic invasion and lymph node metastasis [38]. In another study, CDC25B was more frequently found in patients with deeper tumor invasion and lymph node metastasis in squamous cell carcinomas of the esophagus [20]. CDC25B and CDC25C are thought to be regulators of the G2-M transition through their ability to dephosphorylate and activate the cdk1/CDC2-cyclin B mitotic kinase complex, which is required for cell entry into mitosis [4]. The upregulation of Cdc2 has been found to enhance cell migration, and was correlated with a more motile phenotype of cancer cells [39,40]. The ectopic expression of Cdc2 could increase cell migration *via *a specific association with cyclin B2 and its downstream effector caldesmon, which binds to actin [39,40]. This event could lead to changes in actin filament dynamics and subsequent cell migration. Additionally, integrin and PFTK1 have been shown to promote cell invasion and migration through CDC2 [39,41]. By analogy, we postulate that CDC25B may function in a similar manner in the modulation of HCC cell motility.

## Conclusion

Taken together, our data provide evidence that CDC25B may be a potential target for the clinical management of HCC: it is significantly over-expressed in HCC compared to non-tumor liver; the specific silencing of CDC25B expression inhibited HCC cell growth *in vitro *and *in vivo*, and also inhibited HCC cell migration and invasion *in vitro*. These observations are consistent with other reports [23], and further provide rationale for the development of specific CDC25B small molecule inhibitors as novel agents for cancer treatment [23,42-47]. However, these small molecule inhibitors are often not selective due to considerable structural similarity of the active site regions of the CDC25 family of proteins. Based on our encouraging data with CDC25B specific siRNA, we propose that siRNA may offer a new approach to targeting CDC25B expression and activity in HCC and other malignancies, leading to inhibitions of tumor cell growth and invasion.

## Methods

### Cell culture

The human HCC cell lines Hep3B and Hep40 were purchased from ATCC (Manassas, VA). The cells were cultured in Dulbecco's Modified Eagle's Medium (DMEM) (ATCC, Manassas, VA), supplemented with 10% fetal bovine serum (FBS) (Invitrogen, Carlsbad, CA), 100 μg/ml penicillin and 100 μg/ml streptomycin. Cells were maintained at 37°C in a humidified atmosphere with 5% CO^2^.

### Semi-quantitative real-time PCR

Quantification of CDC25B transcript (from both patient specimens and cell lines) was performed using the MX3000P Real-Time PCR System (Stratagene, La Jolla, CA) as previously described [48]. Human CDC25B (Assay ID: Hs01550934_m1) and 18s rRNA (normalization control) primers and probe reagents were purchased as Pre-Developed TaqMan Gene Expression Assay reagents from Applied Biosystems (Foster City, CA, USA). Transcript quantification was performed in at least duplicate for every sample. The amount of CDC25B was normalized with 18s rRNA to control for RNA amount variation. Paired (HCC and adjacent non-tumor liver) tissues were obtained from 24 patients who underwent liver resection for HCC. This study was approved by the Institutional Review Board for the use of human subjects in medical research, and informed consent was obtained from patients prior to liver resection.

### Immunohistochemistry

Immunohistochemistry was performed using DAKO Envision Plus Kit. (Dako, Carpinteria, CA, USA). Rabbit polyclonal affinity-purified antipeptide antisera against CDC25B were generated by Applied Genomics Inc. (Sunnyvale, CA, USA). We used four tissue microarrays for this study, which consisted of archived tissues retrieved from surgical pathology files. In total, there were 42 normal livers and 248 malignant hepatocellular carcinomas, among other control tissues. Tissue arrays were constructed as previously described [49] with core sizes ranging from 0.6 to 2 mm on different arrays. Arrays were scored using a four-tier scale: 0 – negative (no staining), 1 – weak signal (weak staining in < 50% of cells), 2 – intermediate signal (weak staining in ≥ 50% or strong staining in < 50% of cells), 3 – strong signal (strong staining in ≥ 50% of cells).

### Small interfering RNA transfection

CDC25B-specific siRNAs and Silencer negative control #1 (siRNA-N) were purchased from Ambion, Inc. (Austin, TX). The sequences for CDC25B-specific siRNAs are:

siRNA1-CDC25B (GCCGGAUCAUUCGAAACGATT),

siRNA2-CDC25B (GGAAAAGGACCUCGUCAUGTT),

siRNA3-CDC25B (GCUCUUACUCUUUCCUAUUTT).

The transfection reagent RNAiMAX (Invitrogen, Carlsbad, CA) was used to transfect these siRNAs into Hep3B and Hep40 cells, using the reverse transfection method according to the manufacturer's instructions.

### Western blotting

Transfected cells were harvested and lysed in PARIS lysis buffer purchased from Ambion, Inc. (Austin, TX). Immunoblotting was done with rabbit polyclonal antibody against CDC25B at 250 fold dilution. (Applied Genomics, Inc., Burlingame, CA).

### Cell proliferation assay

For the assessment of cell growth, Hep3B and Hep40 cells transfected with 50 nM siRNA2-CDC25B or siRNA-N were plated on a 96-well plate in triplicate wells. Cell growth was determined by the CellTiter 96 Aqueous One Solution Cell Proliferation Assay (Promega, Madison, Wisconsin, USA) according to the manufacturer's protocol. Optical density (OD) was read at 490 nm at various time points using a SAFIRE microplate reader (TECAN, Research Triangle Park). Once cells have attached (about 4 hours after transfection), a background OD value was obtained; the corresponding background values were then subtracted from data obtained from each well. Three independent experiments were done.

### Invasion/migration assays

Invasion assays were done using the BD Biocoat Matrigel chamber in 24-well plates (BD Biosciences Labware, Bedford, MA). Transfected cells were added to coated filters (5 × 10^4 ^cells/filter) in 500 μl of serum-free DMEM in triplicate wells. In the lower compartments of the chambers, a volume of 750 μl of chemoattractant (DMEM with 20% FBS) was added. After 48 hours incubation at 37°C in a 5% CO^2 ^incubator, the non-invading cells on the upper surface of the filter were wiped off using a cotton swab. Cells that invaded through the filters were fixed, stained with Diff-Quik Stain Set (Dade Behring Inc., Newark, DE, USA), and counted under the microscope by randomly selecting 6 fields per filter (×100 magnification). The migration assay was done in a similar manner but without the Matrigel coating on the filters. Two independent experiments were done for each assay.

### Cell cycle analysis

Cells were transfected with Silencer negative control siRNA-N and siRNA2-CDC25B and then plated on 12-well chamber. Two days after transfection, the floating cells were collected and the adherent cells were detached by trypsin treatment. The floating and detached cells were combined, washed twice with PBS containing 1% BSA, and then were exposed to 70% ethanol at 4°C overnight. After resuspending in PBS containing 1% BSA, cells were subsequently digested by RNase in PBS (100 μg/mL) for 30 min on 37°C, and then stained with propidium iodide (PI) (50 μg/ml in PBS). After incubation for 30 min on ice in the dark, cells were analyzed by flow cytometry (BD FACScan). Cell cycle data were analyzed by Flowjo software.

### Xenograft experiments

All animal experiments were approved by the Administrative Panel on Laboratory Animal Care of Stanford. Ten nude mice (Athymic *nu/nu*, Taconic, NY), ages 4 to 6 weeks (about 20 g of weight) were randomly divided into two groups. Hep40 cells were harvested and mixed with an equal volume of Matrigel (BD Biosciences, Bedford, MA). Cells (10 million) were inoculated into the flank of nude mice. After 50 days, when the average tumor volume (*V*) reached 200 mm^3 ^in each group, the mice were given intratumoral injections of 5 μg siRNA2-CDC25B and RNAiMAX in 30 μl PBS or RNAiMAX alone in 30 μl PBS (as control) every two days. Growth curves were plotted using average tumor volume within each experimental group at the set time points. The tumor dimensions were measured before each injection using a digital caliper, and the tumor volume calculated using the formula: *V *= π/6 × larger diameter × (smaller diameter)^2^.

### Statistical analysis

The statistical analyses were aided by the SPSS version 15.0 software package (SPSS Inc., Chicago, IL, USA). Statistical significance was determined by one-way ANOVA, independent-samples T-Test, or Fisher's Exact Test. *P *values of less than 0.05 and 0.01 were considered statistically significant and highly significant, respectively.

## Competing interests

The author(s) declare that they have no competing interests.

## Authors' contributions

XY contributed to the major part of experimental work, interpreted the results, performed the statistics and drafted the manuscript. MC conceived the study, participated in its design, and contributed with scientific discussion and manuscript preparation. JH ran Q-PCR of tumor samples and analyzed Q-PCR data. SKS is the principal investigator, responsible for conception of the project, designing the experiments, and approving the final manuscript. All authors read and approved the final manuscript.
